# Adverse childhood experiences and the cardiovascular health of children: a cross-sectional study

**DOI:** 10.1186/1471-2431-13-208

**Published:** 2013-12-17

**Authors:** Chelsea Pretty, Deborah D O’Leary, John Cairney, Terrance J Wade

**Affiliations:** 1 Department of Community Health Sciences, Brock University, St Catharines ON, Canada; 2 Departments of Psychiatry and Behavioural Neuroscience, Family Medicine, Kinesiology, CanChild, Centre for Childhood Disability Research, McMaster University, Hamilton ON, Canada; 3 Health Systems Research and Consulting Unit, Centre for Addiction & Mental Health, Toronto, ON, Canada

## Abstract

**Background:**

Adverse childhood experiences (ACEs), such as abuse, household dysfunction, and neglect, have been shown to increase adults’ risk of developing chronic conditions and risk factors for chronic conditions, including cardiovascular disease (CVD). Much less work has investigated the effect of ACEs on children’s physical health status that may lead to adult chronic health conditions. Therefore, the present study examined the relationship between ACEs and early childhood risk factors for adult cardiovascular disease.

**Methods:**

1 234 grade six to eight students participated in school-based data collection, which included resting measures of blood pressure (BP), heart rate (HR), body mass index (BMI) and waist circumference (WC). Parents of these children completed an inventory of ACEs taken from the Childhood Trust Events Survey. Linear regression models were used to assess the relationship between experiencing more than 4 ACEs experienced, systolic BP, HR, BMI and WC. In additional analysis, ACEs were assessed ordinally in their relationship with systolic BP, HR, and BMI as well as clinical obesity and hypertension status.

**Results:**

After adjustment for family education, income, age, sex, physical activity, and parental history of hypertension, and WC for HR models, four or more ACEs had a significant effect on HR (b = 1.8 bpm, 95% CI (0.1-3.6)) BMI (b =1.1 kg/m^2^, 95% CI (0.5-1.8)), and WC (b = 3.6 cm, 95% CI (1.8-5.3)). A dose–response relationship between ACE accumulation and both BMI and WC was also found to be significant. Furthermore, accumulation of 4 or more ACEs was significantly associated with clinical obesity (95^th^ percentile), after controlling for the aforementioned covariates.

**Conclusions:**

In a community sample of grade six to eight children, accumulation of 4 or more ACEs significantly increased BMI, WC and resting HR. Therefore, risk factors related to reported associations between ACEs and cardiovascular outcomes among adults are identifiable in childhood suggesting earlier interventions to reduce CVD risk are required.

## Background

Adverse childhood experiences (ACEs) encompass many possible traumatic and distressing experiences that occur in childhood. Such experiences include traumas such as abuse or neglect but may also include experiences of illness, injury, loss or separation, witnessing a serious event, experiencing a natural disaster and significant changes in the home environment. Research has identified an association between ACEs, such as abuse, household dysfunction, and poverty, and an increased likelihood of developing future health risk factors such as smoking, alcohol and drug use, physical inactivity, and obesity, as well as future chronic illnesses including cardiovascular, lung and liver diseases, and cancer which are, in part, related to these identified risk factors [1-3]. Work by Goodwin & Stein (2004), support these results showing that adults who had previously experienced childhood physical abuse, sexual abuse or neglect were 3.7 times more likely to develop cardiovascular disease (CVD) compared to others [4]. Stein and colleagues (2010) similarly showed that the accumulation of greater than three ACEs was associated with hypertension among adults [5]. Childhood factors including adverse events, socioeconomic status, illness, and growth patterns have also been linked to physiological differences in adult cardiovascular systems, accounting for 3.2% of variation of intima media thickness of the carotid artery in men and 2.2% variation in women [6]. Although this is a small effect, the fact that it remains significant after such a long latency period underscores its importance to cardiovascular health.

While previous studies have demonstrated a connection between ACEs and adult chronic illness and conditions, the majority of studies have been retrospective. That is, adults have been asked to reflect back on their childhood using an inventory of possible ACEs to cue their memory [1-5,7,8] but see [9,10]. By relying on retrospective data collected several decades after childhood, there may be an over- or under-estimation of exposure to ACEs. Moreover, it does not identify when these negative health consequences may begin.

Much of the literature linking ACEs to adult chronic illnesses and conditions has focused on extreme events such as sexual abuse [10], and other forms of severe abuse and maltreatment [1-5,7-9]. Besides these most extreme ACEs, there is evidence of a cumulative effect, or dose–response relationship among adults between the number of reported ACEs and the prevalence of health risk behaviours and chronic diseases [1-3]. Work by Felitti et al. (1998) supports this idea, noting that adults who reported four or more ACEs had increased risk of ischemic heart disease, cancer, chronic bronchitis or emphysema, history of hepatitis or jaundice, skeletal fractures, and poor self-rated health [1].

There is also growing evidence that ACEs may be related to CVD through the mediating effect of obesity. For example, with respect to obesity-induced hypertension [11,12], ACEs have been linked to both high blood pressure (BP) and obesity among adults [1,2,5,7-10]. While the majority of these studies utilize body mass index (BMI) as the measure of obesity [1,7,8], a study by Thomas et al. (2008) found that certain severe ACEs were associated with adult central adiposity, measured using waist circumference (WC) [9]. This is an important distinction because central adiposity has been shown to be a strong predictor of hypertension and CVD [13]. Should there be an association between childhood obesity, measured using central adiposity, and ACEs, this may suggest greater CVD risk in adulthood as childhood obesity and HBP are linked to adult obesity [14] and HBP [15]. Most importantly, the effect of ACEs on CVD risk factors has not been studied in children. One exception was a study completed by Noll et al., (2007) who prospectively assessed the effect of ACEs on obesity in childhood, adolescence and young adulthood [10]. However, these researchers only found a relationship between exposure to ACEs and obesity status in young adults [10]. Furthermore, the study was only completed on female sexual abuse victims.

The primary objective of this study was to examine children and the relationship between ACEs and early childhood risk factors for adult CVD, specifically BP, BMI and WC. In addition, we examined whether ACEs were associated with resting heart rate (HR), a marker of parasympathetic and sympathetic activity. Elevated resting HR is associated with obesity-related hypertension, which may be due to reduced parasympathetic [11,16-18] and/or heightened sympathetic activity [17,19,20]. As elevated HR is a predictor of both adult hypertension and CVD and is associated with a hyperkinetic circulation seen in hypertension [21], it may provide an early marker for risk of elevated BP. We also assess whether there is a cumulative effect of ACEs exposure on these childhood CVD risk factors as cumulative exposure to ACEs has been previously linked to chronic diseases among adults. In summary, the findings from our investigation surrounding the relationship between ACEs, resting HR, BMI, WC and systolic BP in a community sample of 11–14 year old children are presented.

## Methods

### Sample

The data used in this study came from the Heart Behavioural and Environmental Assessment Team (HBEAT) study. A community sample of adolescents aged 11 to 14 years (grades 6 to 8) and their parents from one school board in Southern Ontario were asked to participate in the study. The estimated population base was approximately 5 800 students across 50 schools. The study was approved by both university and school district research ethics review boards. Informed written consent was obtained from the parent/guardian and verbal assent was obtained from the child in order to participate in the study.

Participation was voluntary with no exclusionary criteria and sampling occurred in two phases. The initial phase occurred in fall 2007 involving 28 of the 50 randomly selected schools based on 2006 community census grouping. The remaining 22 schools were approached in winter 2008. In total, 1 913 children volunteered to undergo school-based assessment which included a number of anthropometric measures (i.e. BMI) and automated BP. As well, these adolescents took a questionnaire package home of which 1 324 (69.2%) parent questionnaires were returned.

### Field-testing protocol and measures

#### Blood pressure and heart rate

BP and HR were measured using automatic oscillometric BP units which calculate BP based on the first and fifth Korotkoff sounds (BPM-300, VSM MedTech Devices Inc., Coquitlam, British Columbia, Canada). This unit has been validated for use in children [22]. Students were taken from class in small groups of 8 to 10 to a quiet location in the school. They were asked to relax and to remain silent with their feet flat on the floor sitting upright for about 15 minutes with their arms resting on a table. After 15 minutes, with their right arm positioned at the midpoint of the sternum, BP cuffs were placed on the child’s left arm with cuff size based on arm size [23]. The automatic BP unit took six independent measures at 1-minute intervals. The first three measurements were done to familiarize the subject with cuff pressurization and were discarded. The last three systolic BP (SBP), diastolic BP and HR measures were averaged. Two manual oscillatory BP measurements via sphygmomanometer were taken in the event of an error reading on the automated machine.

#### Anthropometrics

Anthropometric measurements were taken for each student in a private location following BP testing. Students were asked to remove shoes prior to testing. Height (cm) was measured using a portable stadiometer (STAT 7X, Ellard Instrumentation Ltd., Monroe, WA, USA). Body mass (kg) was measured using a calibrated electronic medical scale (BWB-800S, Tanita Corporation, Tokyo, Japan). BMI was calculated as mass (kg) divided by the height squared (m^2^). WC measures (cm) were taken at the narrowest point of the waist, approximately at the location of the belly button [24]. All measures were taken three times and averaged.

#### Parent questionnaire protocol and measures

Parent questionnaires were sent home with students for their parents to complete. The parent questionnaire included an inventory of ACEs , family income, parental education, and family history of hypertension.

#### Adverse childhood experiences

Child adverse experiences were identified through parent report using an inventory adapted from the Childhood Trust Events Survey (CTES 2.0 – Caregiver Form) (see Additional file 1), a 26-item inventory adapted from the Traumatic Stress Survey (TSS) [25]. Certain CTES events were removed due to limitations set forth by the school board (i.e., sexual and physical abuse and maltreatment). Parents were asked to respond to 15 events that possibly occurred and perceived to continue to cause the child a great amount of worry or unhappiness. Additional space was provided for parents to identify other events that had caused their child significant worry or unhappiness. Where applicable, these were recoded and additional ACEs were created for analysis^a^. ACEs that were included in this study are detailed in Table 1. Reliability and validity have not been established for the CTES [26].

**Table 1:** Sample descriptive statistics (n = 1,234)

**Sample characteristics**	**% or Mean ± SD**
Females (%)	55.0
Child age (years, mean ± SD)	11.8 ± 0.9
Parent history of high blood pressure (%)	17.1
Parent education (%)	
Grade 11 or Less	3.2
Grade 12 or Less	5.6
High school diploma (or GED)	6.6
Partial college/training	19.9
College or University degree	40.8
Graduate or Professional degree	23.9
Family income (mean ± SD)	70 828 ± 31 420
Systolic blood pressure (mmHg, mean ± SD)	93.0 ± 8.7
Heart rate (BPM, mean ± SD))	83.4 ± 11.5
Body mass index (kg/m^2^ , mean ± SD)	20.6 ± 4.2
Waist circumference (cm, mean ± SD))	72.4 ± 11.6
Height (cm, mean ± SD)	154.2 ± 8.9
Godin-Shephard (METs/week, mean ± SD )˚	86.6 ± 64.0
Total ACEs	1.9 ± 1.6
**Proportion of sample reporting ACEs**^**‡ **^**(%)**	
Death of family member (not parent)	41.6
Lost a pet that they really cared for (died, killed, lost)	34.2
Serious illness or injury in the family	20.8
Conflict or serious argument between parents	19.5
Divorce or separation of parents	18.6
At least one night stay in a hospital	12.4
Serious illness or injury	8.1
Separation from parents	6.9
Badly frightened or attacked by an animal	5.0
Saw someone get badly hurt or die suddenly	4.9
Family member or residence was robbed	4.5
Death of a parent	3.1

Note: SD = standard deviation; GED = general educational development; BPM = beats per minute; MET = metabolic equivalent; ACEs = adverse childhood experiences; ^‡^Top 12 ACEs reported. ACEs not listed in this table include: in a bad car accident, experienced a natural disaster, moving residence/school or immigrating, separation from sibling or other close family member, a stay in a foster home, other, death of an extended family member or friend, bullying or significant verbal abuse, witnessing serious conflict not between parents. ˚Godin-Shephard measures leisure time physical activity in a one week period.

#### Covariates

Child sex and age (years) were recorded. Family education was based on the maximum level of parental education achieved by any parent (less than grade 11, grade 12, high school diploma or GED, partial college/training, college/university degree, graduate or professional degree). Household income was calculated using the midpoint value of 14 income categories (under $4 999, $5 000 to $9 999, $10 000 to $14 999, $15 000 to $19 999, $20 000 to $24 999, $25 000 to $29 999, $30 000 to $39 999, $40 000 to $49 999, $50 000 to $59 999, $60 000 to $69 999, $70 000 to $79 999, $80 000 to $89 999, $90 000 to $99 999, $100 000 or more[set at $120 000]) and was treated as a continuous variable. Parental history of hypertension was a dichotomous variable based on parental reporting that identified either parent as having received a diagnosis of hypertension (1) compared to neither parent (0). Child physical activity was measured using the Godin-Shephard Leisure -Time Exercise Questionnaire [27]. This questionnaire was completed by students while they waited for their BP to be measured. Students were asked on average how often they participated in strenuous, moderate and mild exercise within a 7-day period and how often in a week they would sweat from exercising in their leisure time. These results were converted into METs (metabolic equivalent), a measure of energy expenditure, for analysis. The Godin-Shephard has been used to estimate physical activity in children within this age range before and has shown reasonable evidence of reliability and validity [28].

### Statistical analysis

**Figure 1:**
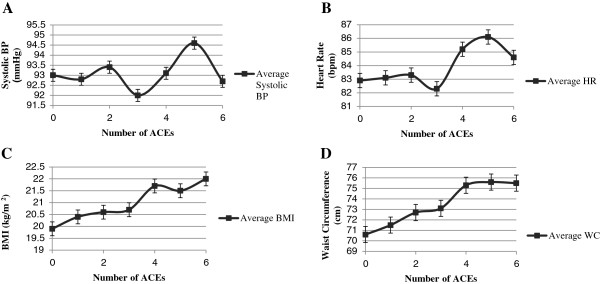
**Average physiological measures by number of ACEs. A** represents the average systolic BP for each ACEs category. **B** represents the average HR for each ACEs category. **C** represents the average BMI for each ACEs category. **D** represents the average WC for each ACEs category. Standard error bars shown. ACEs = Adverse childhood experiences; BP = blood pressure; HR = heart rate; BMI = body mass index; WC = waist circumference.

Any subject with one or more missing variables was removed from the study, reducing the sample size from 1 324 to 1 234. Means and standard errors (SE) of all physiological variables were calculated for each frequency category of exposure to ACEs (Figure 1). Overall, these graphs identified a rise in the mean value of HR, WC and BMI at and above four ACEs. This demonstrated a similar pattern as reported previously by Felitti, et al. (1998) of four or more ACEs being a threshold level [1]. As such, ACEs were coded dichotomously to compare those with fewer than 4 ACEs to 4 or more ACEs. Separate regression analyses were run to test the unadjusted and adjusted effect of dichotomous ACEs on all outcomes. Variables included in the adjusted regression models were child age, sex, physical activity as measured by the Godin-Shepherd, parent history of hypertension, family education level, and family income level. Height was also included in the model for SBP and waist circumference was included in the model for HR.

## Results

This sample included slightly more females than males with students averaging 11.8 years old (Table 1). The majority of families had a parent with at least partial college/training education and an average family income of $71 000. Average SBP in the sample was 93.0 (±8.7) mmHg and mean HR was 83 (±12) beats per minute. On average, the children had a BMI of 20.6 (±4.2) kg/m^2^ with a WC of 72.4 (±11.6) cm. The modal average of ACEs was one, while the mean was approximately two ACEs and 16.0% of the sample experiencing four or more ACEs. Attrition analysis revealed that students who did not have completed parent questionnaires were significantly taller, more likely to be female, more likely to be from a rural or low-income urban school, and had significantly lower HR than the present sample (data not shown).

Table 2 presents the unadjusted linear regression analyses. Having experienced four or more ACEs was found to be significantly associated with higher HR (b = 2.3 bpm, 95% CI (0.6-4.1)), BMI (b = 1.4 kg/m^2^, 95% CI (0.7-2.0)), and WC (b = 3.6 cm, 95% CI (1.8-5.3)). There was no significant effect of ACEs on SBP (b = 0.6 mmHg, 95% CI (−0.7-1.9)). Table 3 adjusts for covariates including family education, family income, parental history of hypertension, and child age, sex, and physical activity. Height was added to the SBP model based on the guidelines set forth by the National High Blood Pressure Education Program Working Group on High Blood Pressure in Children and Adolescents (2004) [29]. Even after the addition of these covariates, the effect of ACEs on BMI (b =1.1 kg/m^2^, 95% CI (0.5-1.8)) and WC (b =3.1 cm, 95% CI (1.3-4.8)) remained significant. Moreover, controlling for these additional covariates as well as for WC (Table 3) the effect of ACEs on HR remained significant (b = 1.8 bpm, 95% CI (0.1-3.6)).

**Table 2:** Unadjusted effect of adverse childhood experiences (ACE) on cardiovascular risk factors

	**SBP b (SE)**	**HR b (SE)**	**BMI b (SE)**	**WC b (SE)**
ACEs (4+ vs <4)	0.6(0.7)	2.3** (0.9)	1.4** (0.3)	3.6** (0.9)
R^2^	0.001	0.055**	0.014**	0.013**

Note: b values are unstandardized regression coefficients. n = 1234. ACE = adverse childhood experiences; BMI = body mass index; HR = heart rate; SBP = systolic blood pressure; SE = standard error; WC = waist circumference *p < 0.05 and **p < 0.01 (two-tailed).

**Table 3:** Adjusted effect of adverse childhood experiences (ACE) on cardiovascular risk factors

	**SBP b (SE)**	**HR b (SE)**	**BMI b (SE)**	**WC b (SE)**
ACEs (≥4 vs <4)	0.34 (0.7)	1.82 (0.9)*	1.13 (0.3)**	3.01 (0.9)**
Child age (years)	−0.24 (0.3)	−1.69 (0.4)**	0.47 (0.1)**	1.55 (0.4)**
Sex	0.84 (0.5)	0.04 (0.7)	0.13 (0.2)	0.56 (0.7)
Height (cm)	0.16 (0.03)**	n/a	n/a	n/a
Waist circumference (cm)	n/a	0.02 (0.03)	n/a	n/a
Godin shephard (METs/week)	−0.005 (0.004)	−0.01 (0.01)**	−0.001 (0.002)	−0.007 (0.01)
Parent history of HBP	2.03 (0.6)**	0.73 (0.9)	0.88 (0.3)**	3.00 (0.9)**
Family education Level	−0.67 (0.2)**	−0.44 (0.3)	−0.32 (0.10)**	−0.85 (0.3)**
Family income (0,000)	−0.1 (0.08)	−0.2 (0.1)	−0.1 (0.04)**	−0.3 (0.1)
Overall model	R^2^ = 0.05**	R^2^ = 0.04**	R^2^ = 0.05**	R^2^ = 0.06**

Note: b values are unstandardized regression coefficients. n/a = not applicable. ACE = adverse childhood experiences; BMI = body mass index; HBP = hypertension; HR = heart rate; MET = metabolic equivalent; SBP = systolic blood pressure; SE = standard error; WC = waist circumference. n = 1234. *p < 0.05 and **p < 0.01 (two-tailed).

Finally, additional regression analyses were completed where ACEs were treated as an ordinal variable as opposed to using the threshold. In these models, both BMI and WC remained significant but HR did not (data not shown). This is not surprising upon further investigation of Figure 1 which reveals a more linear relationship between accumulation of ACEs and both WC and BMI. Regression analyses were also completed with clinically significant outcomes (95^th^ percentile of the sample after adjusting for age, sex, and height for SBP and established cut-offs for BMI) for both BMI and SBP (data not shown) [29,30]. Again ACEs were a significant predictor of obesity at the clinical threshold when assessed both as an ordinal variable and using 4 ACEs as a cut-off. Consistent with prior regression analysis, ACEs were not a significant predictor of hypertension status in the sample.

## Discussion

The present study found a threshold effect in which having experienced 4 or greater ACEs is associated with increased resting HR, BMI and WC in this community sample of 11–14 year old adolescents. There does not appear to be a relationship between ACE and SBP in this sample. Furthermore, there appears to be a dose–response relationship between ACE accumulation, BMI and WC where BMI and WC continue to rise with greater numbers of ACEs.

To the authors’ knowledge, this is the first study to examine the relationship between HR, SBP and ACEs and the second to look at the association between ACEs and obesity in children [10]. Consistent with the work of Felitti et al. (1998) who evaluated the retrospective health effect of reported ACEs in an adult population, examination of the mean physiological measures with each additional ACE (0 through 6) indicated that four or more ACEs appear to be a threshold exposure for children [1]. While this threshold was not related to higher SBP in this study, having experienced four or more ACEs in childhood was linked to a higher HR, BMI, and WC compared to those children who experienced fewer than four events. Nevertheless, this is in contrast to Noll and colleagues (2007) who did not find an association between obesity and certain ACEs until early adulthood [10]. This discrepancy could be the result of the fact that the present study utilized a general population whereas Noll et al. (2007) focused solely on sexual abuse victims [10]. Furthermore, Noll et al. (2007), utilized wider age ranges, assessing obesity in childhood/early adolescence (6–14 years), middle/late adolescence (15–19 years) and young adulthood (20–27 year) [10]. The findings of the current study suggest that the accumulation of ACEs may accelerate obesity as measured by both BMI and WC, and elevate the sympathetic nervous system as indicated by a higher HR. Moreover, it may be that increased BMI and WC precedes any change in BP, which is supported by previous work linking childhood obesity to the development of hypertension in adulthood [15]. Most importantly, this study highlights the novel finding that ACEs have physiological health consequences that begin much earlier than adulthood. Our findings also coincide with the work of Flaherty, et al. (2009) who found an association between five or more ACEs and some indicators of health problems at age 12, including somatic and other health complaints, and illnesses requiring a doctor’s visit [31]. The authors suggested that this age group may be too young to see associations with the negative health behaviours and chronic conditions seen in the ACE Study [1,31]. Our study suggests that we can see the beginnings of these chronic conditions in 11 to 14 year olds. Taken together these studies suggest that ACEs are affecting overall health and prompting physiological changes as early as adolescence.

The significant effect of ACEs on increased HR suggests the existence of a hyperkinetic circulation in these children [20]. Hyperkinetic circulation may be associated with increased sympathetic activity and hypertension in young adults [17,19,20]. There is a current debate in the literature as to whether the elevated HR component of hyperkinetic circulation is driven more by parasympathetic withdrawal [16-19] or by sympathetic over-activation [17,20]. Elevated sympathetic activity is associated with obesity and obesity hypertension. In obesity hypertension, weight gain is seen as the driving force behind elevated BP [11,12]. This weight gain is associated with a rise in sympathetic activity which triggers the renal system to increase sodium retention and therefore, increases blood volume and BP [11,12,32]. Consistent with this, a recent review by Danese & McEwen (2012) focusing on the effect of ACEs on age-related disease [33] proposed that repeated exposure to such events can disrupt the body’s allostatic systems which act to maintain stability through changes in one’s environment [33]. Prolonged engagement of these systems can lead to structural changes in the amygdala in the brain, prolonged activation of the hypothalamic-pituitary-adrenal axis and sympathetic nervous system, as well as, inflammation [33]. These physiological changes may lead to the development of atherosclerosis and subsequently CVD [33]. When the body engages the hypothalamus-pituitary-adrenal response, it secretes hormones to activate the cardiovascular system in order to cope with stress [34,35]. This causes the sympathetic nervous system to increase its involvement in physiological coping [33,35]. HR is initially raised in a hyperarousal state which can persist, or may cause a child to dissociate from the stress [36]. If the trauma is severe and chronic, the resting state for HR and BP are readjusted, resulting in these children living in a physiological heightened state of arousal including higher heart and respiration rates and muscle vigor [36,37]. This physiological remodeling may explain the exposure threshold of four ACEs since HR was significantly elevated among these children, suggesting alterations in neural regulation of the cardiovascular system.

Although the results of this study suggest an earlier effect of ACEs on the cardiovascular system than previously identified, there are some limitations that need to be addressed. First, the three in-school BP readings occurred successively at one point in time. While three measures were taken, we were unable to account for diurnal changes or longer-term variability. Furthermore, without three independent measures at separate time points, no clinical diagnoses can be made. We can only identify those with elevated BP. Second, this study focused primarily on those aged 11 to 14 (grades 6 to 8). This limited age range may have prohibited seeing an effect of ACEs on BP. If BP is in fact affected by ACEs as suggested by Stein, et al., (2010), it may not be apparent until later in adolescence or early adulthood and may be preceded by increased body weight and sympathetic activity [5]. Further research is necessary to evaluate this hypothesis.

This study included a large and diverse community-level sample. This sample was designed to include different segments of the population, including children in urban and rural areas, and in both low and high income areas. Furthermore, a large inventory of possible ACEs was examined in this study to gain an accurate representation of exposure to ACEs in youth. However, some severe ACEs such as physical, sexual and extreme emotional abuse were not examined in this study. The retrospective studies which have noted the dose–response relationship between ACEs and CVD did include these serious events [1-3,5]. Since it was not possible to account for these events, the true impact of ACEs on these measures may be under-estimated. Moreover, the age of the child when such ACEs occurred was not recorded. Differences in timing between the ACE and the measurement of BP may have accounted for this null result. Furthermore, the average income in this sample was quite high, and therefore may not be entirely representative of the general population. Also, the use of the CTES questionnaire as a measure of ACEs could be seen as a limitation as its validity and reliability have not been established in the literature [26].

## Conclusions

In conclusion, this study indicates that in a community sample of grade 6 to 8 children, the accumulation of four or more ACEs was significantly associated with higher BMI, WC and resting HR, as well as obesity status, factors shown to be associated with cardiovascular disease among adults. The findings of this study are very important as they highlight the fact that cardiovascular risks identified among adults exposed to ACEs in previous studies actually begin to appear earlier in childhood. This is a novel finding as Noll and colleagues (2007) only found an association between ACEs and obesity in young adult sexual abuse victims and not children [10]. The current findings emphasize that the physiological consequences of ACEs reported in adults are beginning in childhood. Also, the link between elevated resting HR and ACEs which has not been previously shown may indicate alterations in autonomic regulation that have life-long consequences.

Finally, to gain a better understanding of the relationship between ACEs and BP, research should focus on older adolescents, ideally tracking them over time to evaluate whether the effect on BP occurs in later years and whether it is preceded by changes in BMI and WC. Danese & McEwen argue that the adverse effects of ACEs in childhood can be reversible if the child’s environment is returned to a stable state [33,38,39]. This suggests that studies should examine how these risks can be mitigated among children and whether this can reduce the potential long-term health consequences shown repeatedly in studies among adults.

## Endnote

^a^These ACEs included: death of a close friend or extended family member, moving residence/school or immigration, separation from sibling or other close family member, bullying or significant verbal abuse, and witnessing serious conflict not between parents.

## Abbreviations

ACE: Adverse childhood experience; BP: Blood pressure; BMI: Body mass index; CTES: Childhood trust events survey; CVD: Cardiovascular disease; HBEAT: Heart behavioural and environmental assessment team; HBP: High blood pressure; HR: Heart rate; MET: Metabolic equivalent; SBP: Systolic blood pressure; WC: Waist circumference.

## Competing interests

The authors declare that they have no competing interests.

## Authors’ contributions

CP performed the statistical analysis, participated in the acquisition of data, and drafted the manuscript. DDO and JC participated in design and coordination of the present study and contributed to revising the manuscript. TJW was responsible for the design and coordination of the present study, conceptualization of the analysis, oversight of the statistical analysis, and assisted in drafting the manuscript. All authors read and approved the final manuscript.

## Pre-publication history

The pre-publication history for this paper can be accessed here:

http://www.biomedcentral.com/1471-2431/13/208/prepub

## Supplementary Material

Additional file 1 **The Childhood Trust Events Survey.** Children and adolescents: Caregiver form, Version 2.0 (10/10/2006). The CTES was developed by the Trauma Treatment Training Center, The Childhood Trust & The Mayerson Center for Safe and Healthy Children, Cincinnati Children’s Hospital Medical Center. Click here for file
