# Supplementary material for: Adverse childhood experiences and the cardiovascular health of children: a cross-sectional study
Source: BMC Pediatr. 2013 Dec 17;13:208. doi: 10.1186/1471-2431-13-208 (PMC3878623; doi:10.1186/1471-2431-13-208)
Supplement: Additional file 1 — The Childhood Trust Events Survey. Children and adolescents: Caregiver form, Version 2.0 (10/10/2006). The CTES was developed by the Trauma Treatment Training Center, The Childhood Trust & The Mayerson Center for Safe and Healthy Children, Cincinnati Children’s Hospital Medical Center. [file 1471-2431-13-208-S1.pdf]

ID ____________________

Date ____________________

DOB/age ____________________

The Childhood Trust Events Survey

Children and Adolescents: Caregiver Form

Version 2.0; 10/10/2006

It is important for us to understand what may have happened to your child. The questions below describe some kinds of upsetting experiences. Since we give these questions to everyone, we list a lot of possible events that may have happened at any time in your child's life. If one or more of these experiences has happened at some time in your child's life, please circle **Y** for **Yes**. If not, circle **N** for **No**. If you are unsure, circle **DK** for **Don't Know**. Thank you for completing this survey.

1.	Was your child ever in a really bad accident, such as a serious car accident?	Y	N	DK
2.	Was your child ever in a disaster such as a tornado, hurricane, fire, big earthquake, or flood?	Y	N	DK
3.	Was your child ever so badly hurt or sick that he/she had to have painful or frightening medical treatment?	Y	N	DK
4.	Has your child ever been threatened or harassed by a bully (someone outside of his/her family)?	Y	N	DK
5.	Has your child ever repeatedly had a parent swear at him/her, insult him/her, or had hurtful things said to him/her such as "You are no good," "You will be sent away because you are bad," or "I wish you were never born"?	Y	N	DK
6.	Was your child ever completely separated from his/her parent(s) for a long time, such as going to a foster home, the parent living far apart from him/her, or never seeing the parent again?	Y	N	DK
7.	Has your child ever had a family member who was put in jail or prison or taken away by the police?	Y	N	DK
8.	Has your child ever had a time in his/her life when he/she did not have the right care, such as not having enough to eat, being left in charge of younger brothers or sisters for long periods of time, or being left with an adult who used drugs?	Y	N	DK
9.	Has your child ever had a time in his/her life when he/she was living in a car, living in a homeless shelter, living in a battered women's shelter, or living on the street?	Y	N	DK
10.	Has your child ever had someone living in his/her home who abused alcohol or used street drugs?	Y	N	DK
11.	Has your child ever seen someone in the home try to hurt or kill himself/herself, such as cutting himself/herself or taking too many pills or drugs?	Y	N	DK

Page 1 subtotals ___ ___ ___

12. Has your child ever had a family member who was depressed or mentally ill for a long time?	Y	N	DK
13. Has your child ever had a family member or someone else very close to him/her die unexpectedly?	Y	N	DK
14. Has someone in your child's home ever been physically violent toward him/her, such as whipping, kicking, or hitting hard enough to leave marks?	Y	N	DK
15. Has an adult ever said they were going to hurt your child really badly or kill him/her, or acted like they were going to hurt your child very badly or kill him/her, even if this person didn't actually do it?	Y	N	DK
16. Has your child ever seen or heard family members act like they were going to kill or hurt each other badly, even if they didn't actually do it?	Y	N	DK
17. Has your child ever seen or heard a family member being hit, punched, kicked very hard, or killed?	Y	N	DK
18. Has your child ever seen someone in his/her neighborhood be beaten up, shot at or killed?	Y	N	DK
19. Has someone ever robbed or tried to rob (jump) your child or your child's family with a weapon?	Y	N	DK
20. Has someone ever kidnapped your child or has someone close to your child ever been kidnapped?	Y	N	DK
21. Has your child ever been badly hurt by an animal, such as attacked by a dog?	Y	N	DK
22. Has your child ever had a pet or animal that was hurt or killed on purpose by someone he/she knew?	Y	N	DK
23. Has your child ever seen a friend killed?	Y	N	DK
24. Has someone ever touched your child's private sexual body parts when he/she did not want them to?	Y	N	DK
25. Has someone ever made your child touch another person's private sexual body parts?	Y	N	DK
26. Has an adult ever tied your child up, gagged him/her, blindfolded him/her, or locked him/her in a closet or a dark scary place?	Y	N	DK
Page 2 subtotals	___	___	___
Page 1 subtotals	___	___	___
Total	___	___	___

If more than one event happened AND still seems to bother your child, put a star next to the one that you believe bothers him/her the most.

Trauma Treatment Training Center
The Childhood Trust & The Mayerson Center for Safe and Healthy Children
Cincinnati Children's Hospital Medical Center
3333 Burnet Ave, MLC 3008 Cincinnati, Ohio 45229-3039

This survey is a public domain document and may be freely reproduced and distributed without copyright restrictions. Please do not alter the item wording or content or the response format and then distribute the modified version under the original name. If you feel you must make any modifications of this survey, please rename it so that others will not be confused. For more information about this scale, please contact Erica Pearl, Psy.D. Email: erica.pearl@cchmc.org.
